# Supplementary material for: Continuing to be cautious: Japanese contact patterns during the COVID-19 pandemic and their association with public health recommendations
Source: PLOS Glob Public Health. 2025 Sep 24;5(9):e0004600. doi: 10.1371/journal.pgph.0004600 (PMC12459842; doi:10.1371/journal.pgph.0004600)

SI Text

Table A. List of variables that were included in the social contact survey and analyzed when developing the multivariable regression model using Weibull distribution.

Variable	Response reported in contact survey
Individual and household characteristics	
Age	Numerical value
Sex	Male/Female
Prefecture of residence	Osaka/Fukuoka
Do you or any of your household members belong to a high risk category with health conditions or chronic diseases that hinder your/their everyday life? (e.g. chronic respiratory disease, chronic heart disease, chronic kidney disease, chronic liver disease, and chronic neurological disease	List the person who meets this category
Occupation	List given as shown on Table 2
Number of people in the same household	Numerical value
Health mitigation and COVID-19 related characteristics	
How concerned are you of getting infected with COVID-19?	1: Mostly concerned, 2: Somewhat concerned, 3: Neither concerned nor unconcerned, 4: Somewhat unconcerned, 5: Mostly unconcerned, 6: Don't know, 7: Do not want to answer
Have you or any other household member been tested positive by PCR for coronavirus (COVID-19)?	List the person who meets this category
How long did you wear a mask today?	Numerical value
How many times did you wash your hands with soap in the last 3 hours?	Numerical value
How many times have you been vaccinated against COVID-19?	Numerical value
Work-related characteristics	
Where was your main work location?	Home, Workplace, School, Not employed
How frequently did you telework or attend school classes at home?	0: Never/not possible, 1: A few times per month, 2: 2-3 times per week, 3: 4-5 times per week
During the past month, how many times did you travel to a prefecture outside your home prefecture for work?	Numerical value
Possible locations of contact	
Home	Yes/No
Other person's home	Yes/No
Work	Yes/No
School	Yes/No
Restaurant	Yes/No
Bar/karaoke	Yes/No
Shop	Yes/No
Place of worship	Yes/No
Public transportation	Yes/No
Gym	Yes/No
Movie	Yes/No
Market/Supermarket/Convenience store	Yes/No
Other place	Yes/No

Fig A. Plot showing the overall mean contacts based on various cutoff points for truncated contacts ranging from 10 to 500 contacts. A Weibull distribution was used to describe the mean contacts reported throughout the week from the February 2023 contact survey.

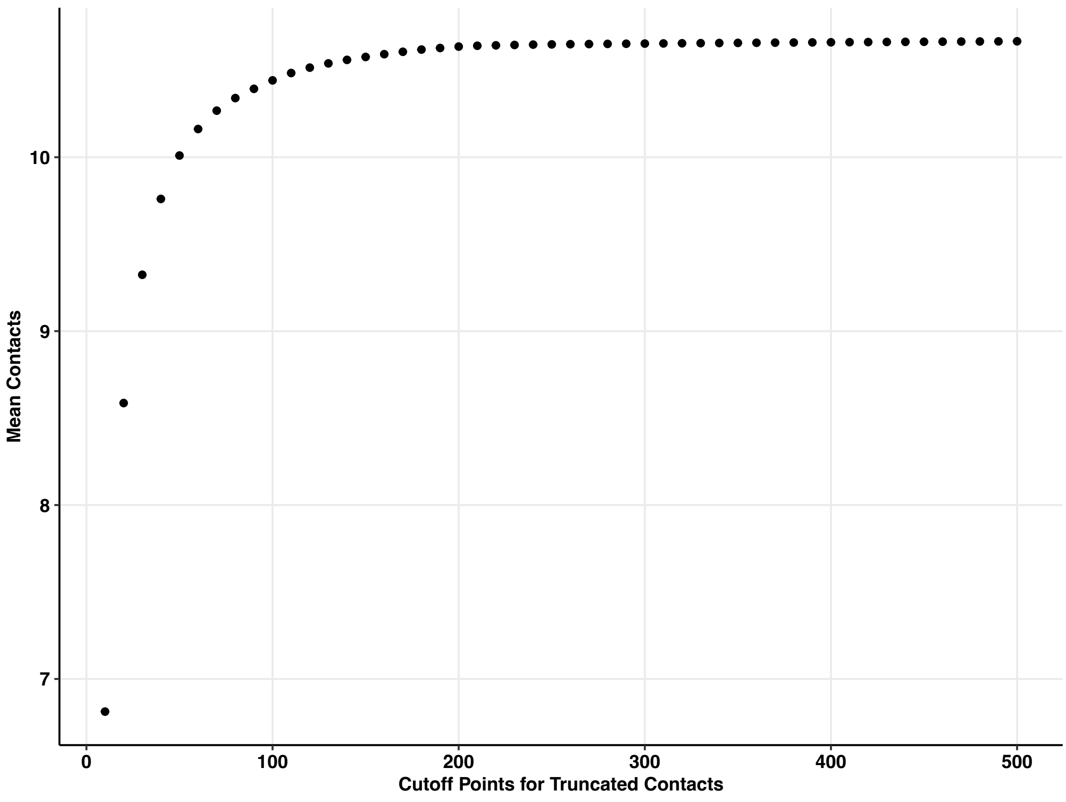

Fig B. A comparison of national data of individuals who received the COVID-19 vaccination as of January 2023 with the individuals who reported their doses of vaccination from the social contact surveys conducted in February 2023.[1]

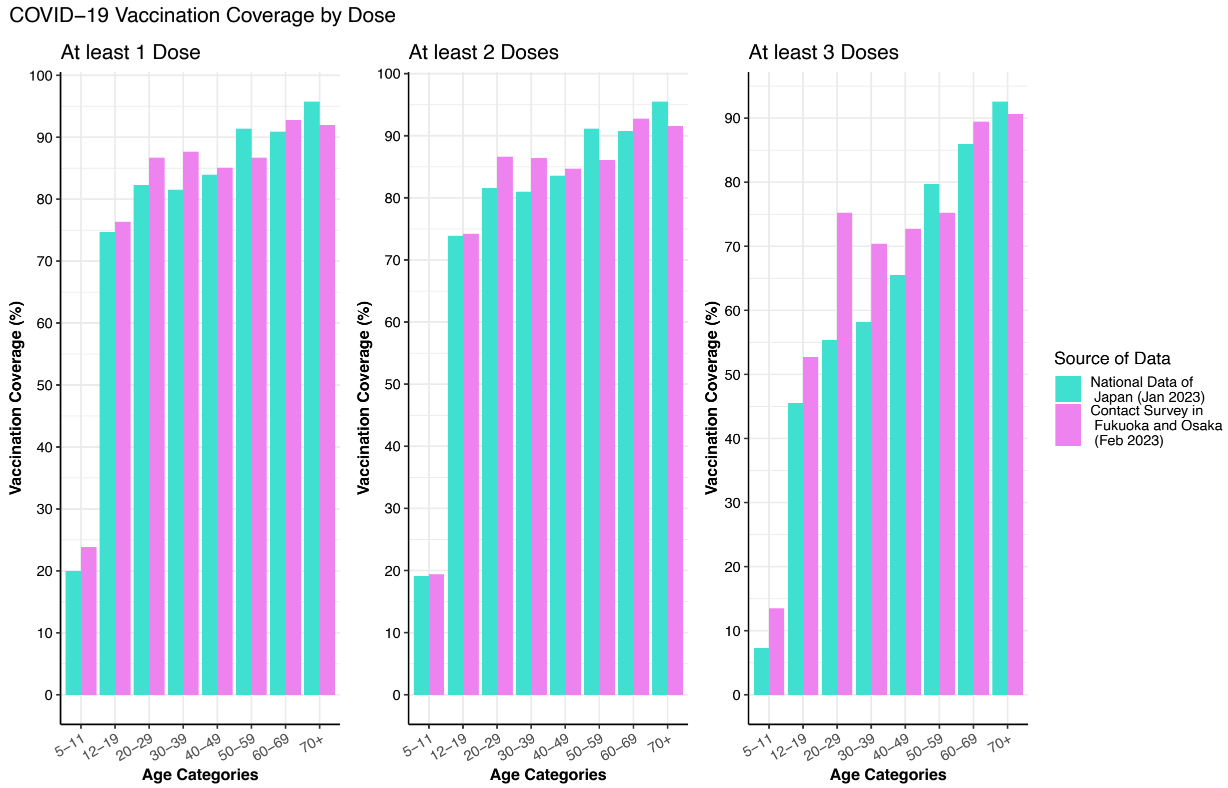

[1] Not all national vaccination coverage data of Japan has been publicly available. However, data from January 2023 collected by the Prime Minister's Office of Japan was the time point that was most comparable with the social contact survey and was shared with permission by the National Institute of Infectious Diseases (Japan).

Fig C. Distribution of contacts reported per individual during the weekday in Fukuoka and Osaka prefectures from February 2021 to February 2023.

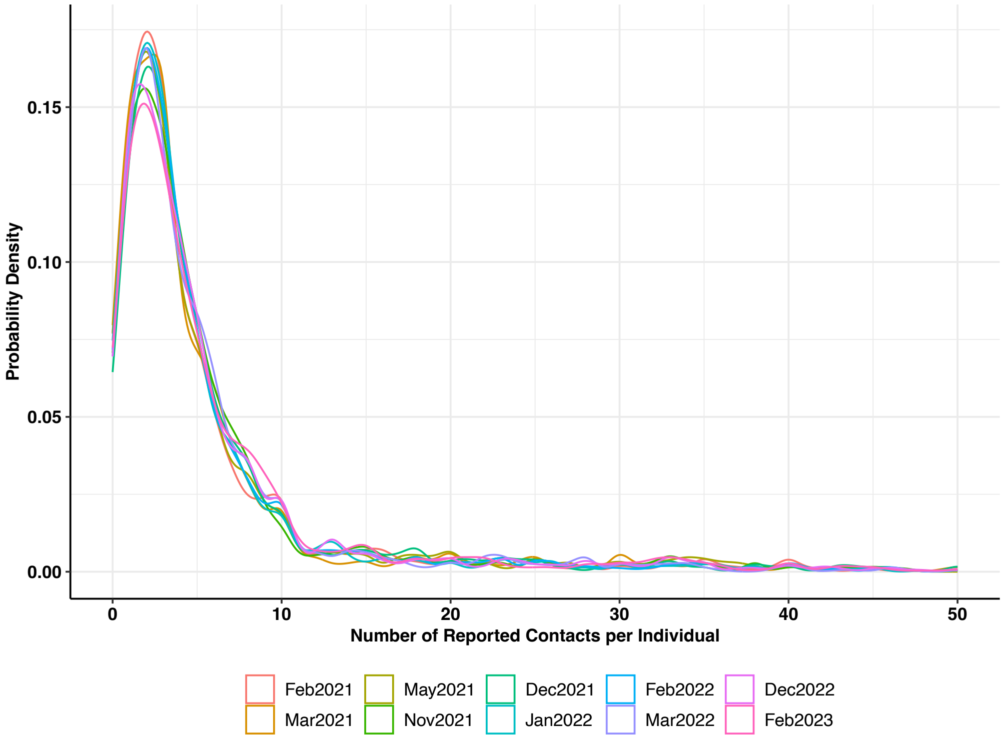

Table B. Timetable of Japan's restrictions, including public health emergency declarations and governmental recommendations, implemented to curb COVID-19 transmission from 2020 to 2023.

Start Date	End Date	Place	Regulation Type/Event	Description
2020-03-02	2020-05 (varied by city/town/village)	National	School closure	All primary, middle and high schools to be closed at least until spring break that starts end of March.
2020-03-21	2022-10-11	National	Border control	Forbidden entry to Japan from abroad (all tourists, business) except for Japanese citizens, permanent residents
2020-04-07	2020-05-14 (Fukuoka) 2020-05-21 (Osaka)	Prefectures including Osaka, Fukuoka	State of emergency	Recommendation include (but not limited to):<br>• Stay at home<br>• Limit social contacts in 3C settings<br>• Discourage moving between prefectures for non-essential travel<br>• Complete closure or shortening of restaurant/bar hours<br>• Restricted hours of serving alcohol
2020-04-16	2020-05-25 (last prefecture)	National	State of emergency	Same as above
2021-01-14	2021-02-28	Prefectures including Osaka, Fukuoka	State of emergency	Same as previous state of emergency
2021-02-17	Ongoing	National	Vaccination	First COVID-19 vaccination to start for healthcare workers
2021-05-17	Ongoing	Tokyo, Osaka	Vaccination	Mass COVID-19 vaccination to start for 65-year-old and above
2021-06-21	Ongoing	National	Vaccination	Mass COVID-19 vaccination for the public to start at universities and workplace
2021-04-24	2021-06-19	Tokyo, Osaka, Hyogo, Kyoto	State of emergency	Same as previous state of emergency
2021-05-11	2021-06-19	Prefectures including Fukuoka	State of emergency	Same as previous state of emergency
2021-07-11	2021-09-30	Tokyo	State of emergency	Same as previous state of emergency
2021-07-23	2021-08-08	Tokyo	Summer Olympics	No general audience during the Olympics
2021-08-02	2021-09-30	Prefectures including Osaka	State of emergency	Same as previous state of emergency
2021-08-20	2021-09-30	Prefectures including Fukuoka	State of emergency	Same as previous state of emergency
2022-01-27	2022-03-21	Prefectures including Osaka, Fukuoka	State of semi-emergency	Soft recommendation to limit social contacts in 3C settings, shortening of restaurant/bar hours. No stay-at-home and closure of business policies.

Fig D. A series of residual plots from the multivariable regression model using a Weibull distribution based on weekday contacts in Feb 2023. **1.** Residual plot showing predicted contacts in natural scale. **2.** Residual plot showing predicted contacts in a log link function. **3** through **6** show residuals against each of the selected predictors in the multivariable regression model.

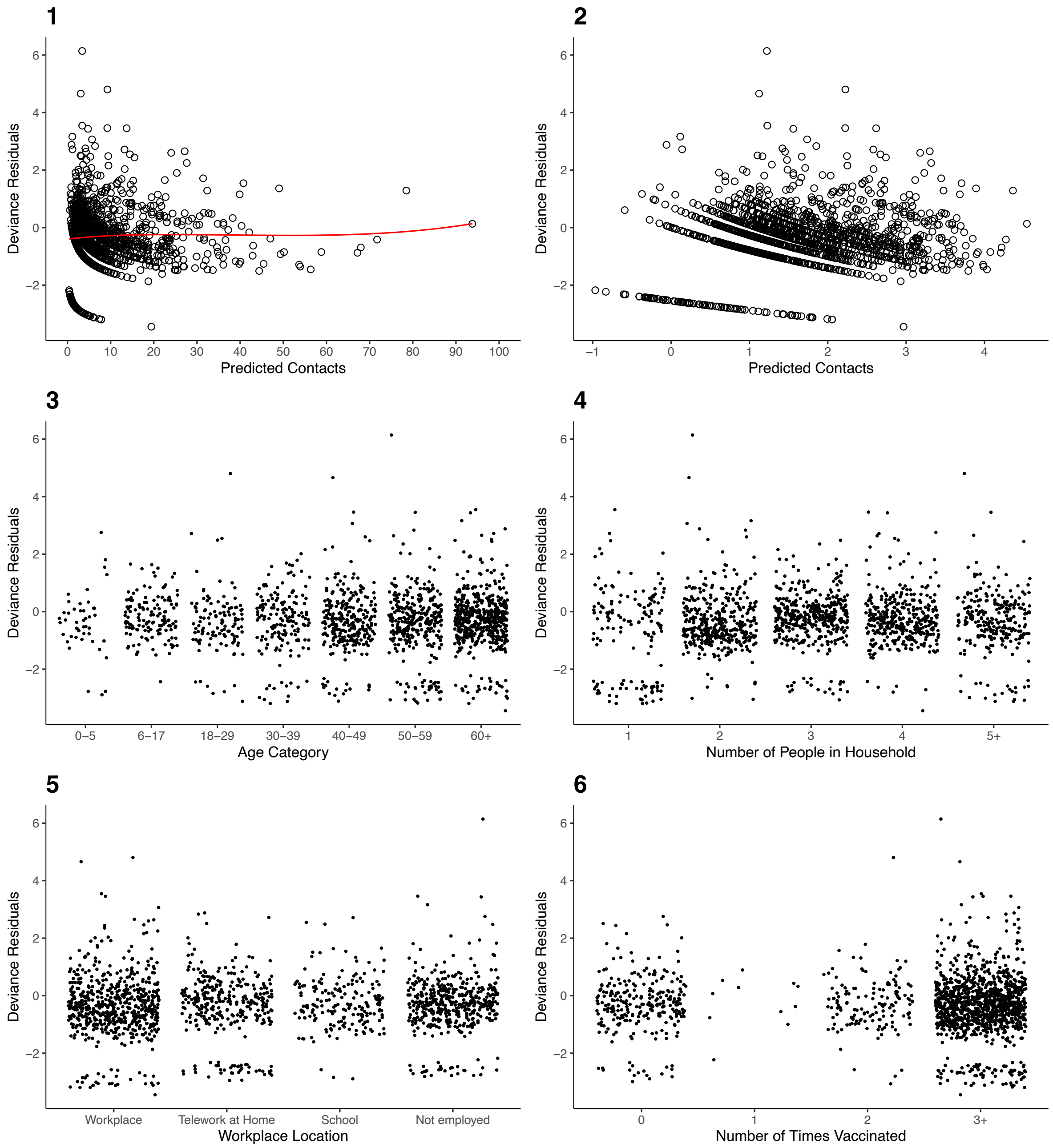

Table C. Covariates used in the multivariable regression model that showed multicollinearity with a Variance Inflation Factor (VIF) higher than 2. Other covariates, including other categories of occupation, that are not listed here had VIFs lower than 2.

Category	VIF
**Participant Age**	
0-5	>5
6-17	>5
18-29	2.09
30-39	1.49
40-49 (reference)	N/A
50-59	1.82
60+	2.56
**Number in Household**	
1 person	2.06
2 people	1.57
3 people	N/A
4 people	1.59
5+ people	1.56
**Occupation**	
Freelancer	4.48
Kindergartner	>5
Primary school student	>5
Middle school student	>5
High school student	>5
Post high school prep student	3.74
Unemployed/helps at the house	2.26
Pension retiree	2.53
Other	4.47
**Location of Work**	
Workplace (reference)	N/A
Telework	2.45
School	>5
Not Employed	>5
**Reported Location of Contact**	
School	3.15
**Perspective on COVID-19**	
1 – Most concerned (reference)	N/A
2 - Concerned	1.79
3 - Neutral	1.47
4 - Not concerned	1.47
5 - Least concerned	1.31
6 - Do not know	>5

Fig E. The mean number of weekday contacts was calculated based on the first ten contacts that were reported by the study participants in Fukuoka and Osaka prefectures from February 2021 to February 2023 for physical contacts **(1)** and non-physical contacts **(2)**. A contact is defined here as any contact that occurred at an indoor setting (includes contacts that could have happened both indoor AND outdoor). The mean and 95% confidence intervals are obtained by bootstrapping.

Fig E.1. Mean Number of Physical Contacts during the Weekday

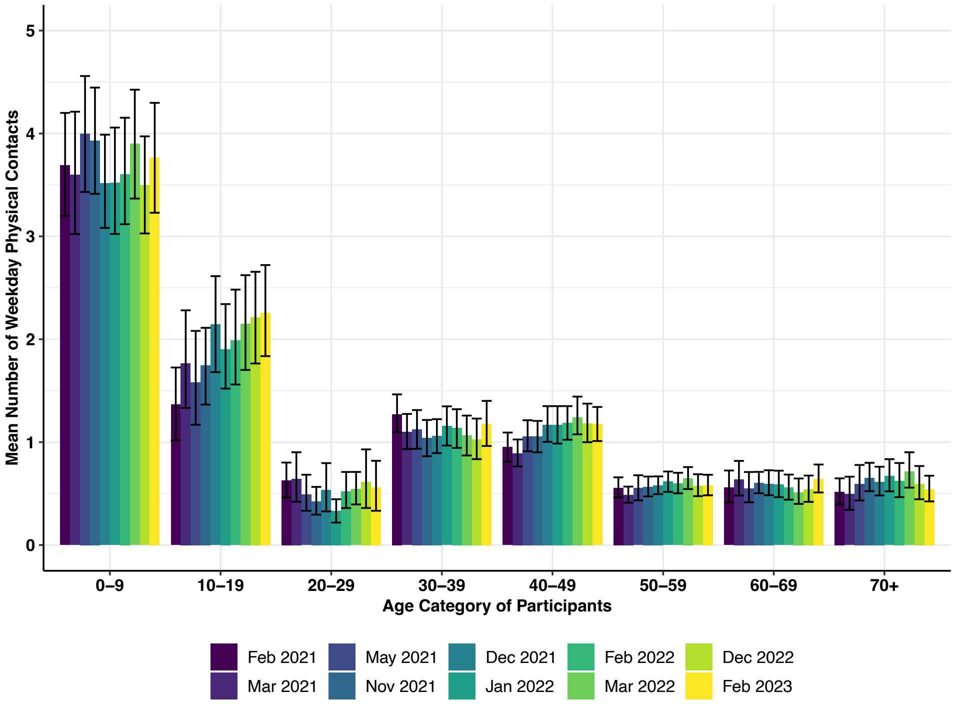

Fig E.2. Mean Number of Non-Physical Contacts during the Weekday

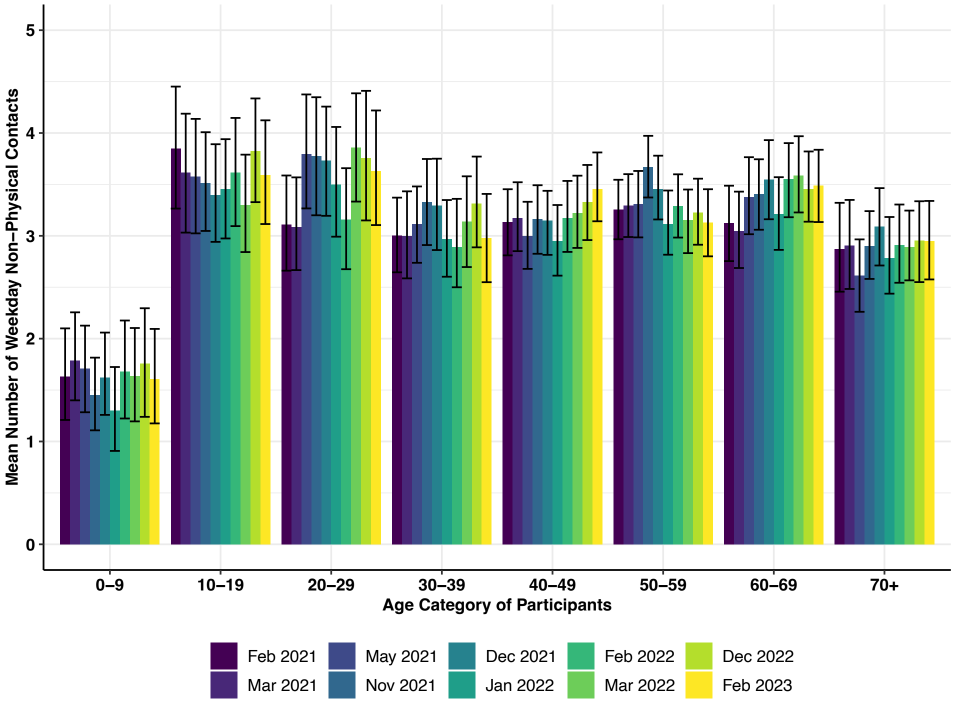

Fig F. The mean number of weekday contacts was calculated based on the first ten contacts that were reported by the study participants in Fukuoka and Osaka prefectures from February 2021 to February 2023 that took place indoors **(1)** and outdoors **(2)**. A contact is defined here as any contact that occurred at an indoor setting (includes contacts that could have happened both indoor AND outdoor). The mean and 95% confidence intervals are obtained by bootstrapping.

Fig F.1. Mean Number of Weekday Indoor Contacts

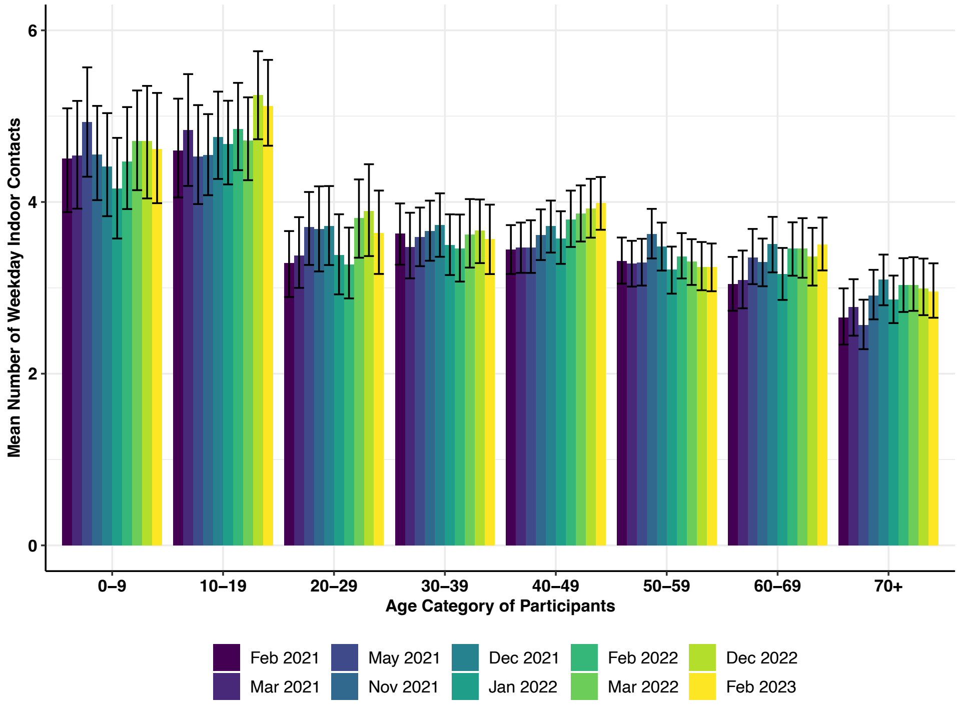

Fig F.2. Mean Number of Weekday Outdoor Contacts

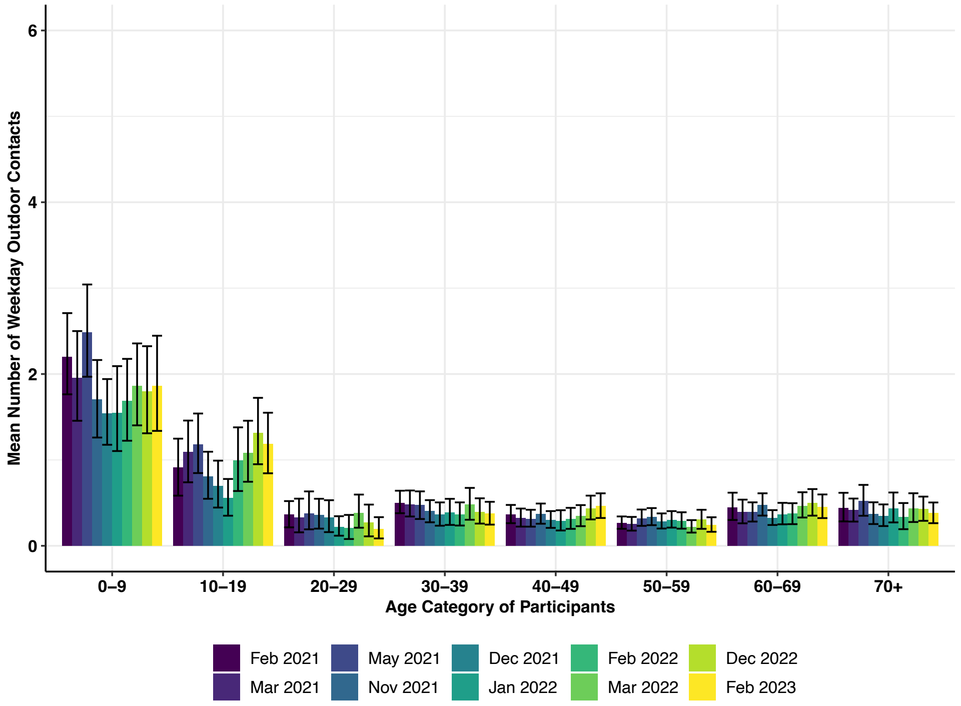

Supplement: S1 Text — (PDF) [file pgph.0004600.s001.pdf]
